# Reversine-Induced Telomere Architecture Remodeling in Chronic Myeloid Leukemia Cell Lines: Insights from TeloView^®^ Analysis of 3D Nuclear Architecture

**DOI:** 10.3390/cimb47110907

**Published:** 2025-10-31

**Authors:** Fábio Morato de Oliveira, Isabela Dias Cruvinel, Bruno Machado Rezende Ferreira, Sabine Mai

**Affiliations:** 1 Laboratory of Human and Medical Genetics, Federal University of Jataí, Jataí 75801-615, Goiás, Brazil; 2 Department of Physiology and Pathophysiology, University of Manitoba, Winnipeg, MB R3E 0J9, Canada

**Keywords:** chronic myeloid leukemia, reversine, telomeres, TeloView^®^, genomic instability, aurora kinases

## Abstract

Reversine is a small-molecule Aurora kinase inhibitor known for its pro-apoptotic effects and potential to remodel chromatin architecture. Although its impact on mitotic regulation is established, its effects on telomere dynamics and nuclear organization in chronic myeloid leukemia (CML) remain unclear. This study aimed to investigate the effects of reversine on telomere architecture, genomic instability, and apoptosis in CML cell lines (K-562 and MEG-01). Reversine was applied at increasing concentrations, and cytotoxicity was assessed using caspase-3/7 activation assays. Quantitative PCR was used to measure *AURKA* and *AURKB* mRNA expressions. Three-dimensional telomere architecture was analyzed with TeloView^®^ v1.03 software after Q-FISH labeling to quantify telomere number, signal intensity, aggregation, nuclear volume, and *a/c ratio*. Reversine induced a dose- and time-dependent apoptotic response in both cell lines and significantly downregulated *AURKA* and *AURKB* expressions. Three-dimensional telomere analysis revealed a marked reduction in telomere number and aggregates, signal intensity, and nuclear volume. While reduced signal intensity may indicate telomere shortening, the concurrent decrease in aggregation and altered spatial parameters suggests telomeric reorganization rather than progressive instability. These features reflect structural nuclear remodeling and early apoptotic commitment. Differences between K-562 and MEG-01 responses underscore potential heterogeneity in telomere maintenance mechanisms. Reversine modulates genomic stability in CML cells through dual mechanisms involving Aurora kinase inhibition and telomere architecture remodeling. The integration of 3D telomere profiling highlights reversine’s potential as a therapeutic agent targeting nuclear disorganization and mitotic dysregulation in leukemia.

## 1. Introduction

Reversine is a small synthetic purine analog that has gained attention for its dual role in cellular dedifferentiation and inhibition of mitotic kinases [1,2,3]. Initially identified as an agent capable of inducing dedifferentiation of committed cells into multipotent progenitor-like cells [2,3], reversine has since been recognized for its inhibitory effects on aurora kinases, particularly aurora A and B, which are crucial regulators of mitosis [4]. By targeting these kinases, reversine disrupts mitotic progression, leading to cytokinesis failure, polyploidization, and suppression of tumor cell proliferation [5].

Aurora kinases, including *AURKA* and *AURKB*, are serine/threonine kinases that regulate chromosome segregation and cytokinesis during cell division [5]. Overexpression of these kinases has been implicated in some cancers, including hematological malignancies [5,6,7,8]. In chronic myeloid leukemia (CML), the *BCR-ABL* oncoprotein upregulates *AURKA* and *AURKB* via the Akt signaling pathway, promoting leukemogenesis [9]. Elevated expression of these kinases has been correlated with poor prognosis and resistance to tyrosine kinase inhibitors (TKIs), which are the standard treatment for CML [10].

Several studies have demonstrated that reversine inhibits leukemia cell proliferation by targeting aurora kinases. Research on acute myeloid leukemia (AML) cells found that reversine significantly reduced colony formation and induced apoptosis, while displaying lower toxicity toward healthy donor cells compared to other aurora kinase inhibitors such as VX-680 [2]. Additionally, reversine has been found to cause mitotic defects and polyploidization, resulting in the suppression of glioma cells and other tumors [11,12]. Studies have also reported that reversine inhibits the proliferation of chronic myeloid leukemia (CML) cells, supporting its potential as an anti-leukemic agent [13]. This selective cytotoxicity suggests that reversine may be a promising candidate for leukemia therapy, including in CML, where dysregulation of aurora kinases plays a role in disease progression [13,14,15].

Three-dimensional (3D) telomere analysis, performed using TeloView^®^ software, enables quantitative assessment of the 3D telomere architecture by evaluating parameters such as telomere number, length, signal intensity, spatial distribution, and the presence of telomere aggregates [16,17]. In CML, abnormalities in telomere dynamics have been correlated with disease progression, with increased telomere aggregates and nuclear volume alterations being observed in advanced phases of the disease [18]. Studies have shown that as CML progresses from the chronic phase to the blast crisis, there is a shift in telomere organization, which may be linked to genomic instability and resistance to therapy [19].

Although direct studies on reversine’s impact on 3D telomere architecture in CML cells remain unknown, its role as an aurora kinase inhibitor suggests a potential influence on telomere maintenance. Aurora kinases regulate mitotic progression and chromosome segregation, both of which are crucial for telomere stability [20,21]. The inhibition of aurora kinases by reversine has been associated with mitotic defects, aneuploidy, and altered nuclear architecture, which could indirectly affect telomere dynamics [19,22].

In this study, we investigated the effects of reversine on mitotic progression and genomic stability in chronic myeloid leukemia (CML) cell lines. Specifically, we analyzed the impact of reversine on 3D telomere architecture in MEG-01 and K-562 cells using TeloView^®^ v1.03 software, focusing on parameters such as telomere number, length, aggregation, and spatial organization. In parallel, we assessed the expression of aurora kinase genes (*AURKA* and *AURKB*) to explore how reversine modulates mitotic regulators. This approach allowed us to evaluate the compound’s potential to alter nuclear architecture and induce apoptosis in CML cells through mechanisms linked to mitotic disruption and telomere dysfunction.

## 2. Material and Methods

### 2.1. Cell Culture

For this study, MEG-01 and K-562 cells, obtained from American Type Culture Collection (ATCC, Manassas, VA, USA), were cultured in RPMI-1640 medium supplemented with 10% fetal bovine serum (FBS), 1% L-glutamine, and 1% penicillin-streptomycin (Thermo Fisher Scientific, Waltham, MA, USA). The cells were maintained at 37 °C in a humidified atmosphere with 5% CO_2_. To investigate the effects of reversine (Cayman Chemical, Ann Arbor, MI, USA), a selective aurora kinase inhibitor, the compound was dissolved in dimethyl sulfoxide to prepare a 10 mM stock solution (DMSO; Sigma-Aldrich, St. Louis, MO, USA). Working concentrations of 5 µM, 10 µM, 20 µM and 30 µM were freshly prepared, and cells were treated for 24, 48 and 72 h, with control groups receiving an equivalent amount of DMSO (<0.1% *v*/*v*).

### 2.2. Cytotoxicity Assessment and Cell Viability Analysis

To investigate the mechanism of reversine-induced apoptosis in chronic myeloid leukemia (CML) cell lines, caspase-3/7 enzymatic activity was assessed using the Caspase-Glo^®^ 3/7 Assay System (Promega, Madison, WI, USA), following the manufacturer’s instructions. K-562 and MEG-01 cells were seeded in 96-well plates at a density of 1 × 10^4^ cells per well in 100 μL of culture medium. Cells were treated with increasing concentrations of reversine (5, 10, 20, and 30 µM) for 24, 48, and 72 h. Control wells received vehicle (DMSO) at a final concentration of <0.1%. After treatment, 100 μL of Caspase-Glo^®^ 3/7 reagent was added directly to each well (1:1 volume ratio). Plates were gently mixed for 30 s and incubated at room temperature in the dark for 1 h. Luminescence, which is proportional to caspase-3/7 activity, was measured using a microplate luminometer (e.g., GloMax^®^ Discover System (Promega Corporation, Madison, WI, USA). Luminescence values were normalized to DMSO-treated control and expressed as percentage of apoptotic signal relative to the highest observed response. All experimental conditions were performed in triplicate, and each assay was independently repeated at least three times to ensure reproducibility. Data are presented as mean ± standard deviation (SD) from three independent experiments unless otherwise stated. Statistical comparisons between treated and control groups were made using one-way ANOVA followed by Bonferroni post hoc tests, with *p*-values < 0.05 considered statistically significant. GraphPad Prism 8 (GraphPad Software, San Diego, CA, USA) software was used for all analyses. The half-maximal inhibitory concentration (DL_50_) was calculated using non-linear regression analysis in GraphPad Prism 8.

### 2.3. Quantitative Fluorescent In Situ Hybridization (Q-FISH)

For evaluation of telomere architecture before and after reversine treatment, the cells were harvested and fixed with 4% paraformaldehyde. Fixed cells were then placed onto poly-L-lysine-coated coverslips, and nuclei were counterstained with 4′,6-diamidino-2-phenylindole (0.1 µg/mL) (DAPI; Thermo Fisher Scientific, Waltham, MA, USA) before being mounted with an antifade reagent. Telomere staining was performed using Cy3-labeled telomeric peptide nucleic acid (PNA) probes (Thermo Fisher Scientific, Waltham, MA, USA), following a standard Quantitative Fluorescence in situ Hybridization (Q-FISH) protocol. The hybridization process involved denaturation at 82 °C for three minutes, followed by incubation at 30 °C for two hours. Post-hybridization washes were carried out using SSC buffer and PBS to remove non-specific binding.

### 2.4. D Image Acquisition and Processing

Interphase nuclei from each sample were analyzed using an AxioImager M1 microscope (Carl Zeiss, Jena, Germany) equipped with an AxioCam HRm charge-coupled device and a 63× oil objective lens (Carl Zeiss, Jena, Germany). Acquisition times were 500 ms for Cy3 (telomeres) and 5 ms for DAPI (nuclei). Sixty z-stacks were captured at a sampling distance of x, y: 102 nm and z: 200 nm for each stack slice. Images were deconvolved and converted to TIFF files for 3D analysis using TeloView^®^ v1.03 software (Telo Genomics Corp., Toronto, ON, Canada) software [16,17].

### 2.5. TeloView^®^ Analysis and Statistics

To compare telomere architecture among treated samples, the TeloView^®^ software, proprietary to Telo Genomics, Toronto, Canada, was used [16,17]. For each experimental condition, a minimum of 50 interphase nuclei were analyzed to ensure sufficient statistical power for telomere parameter quantification. TeloView^®^ measures six distinct parameters: (1) telomere length based on signal intensity, (2) the number of telomere signals per nucleus, (3) the number of telomeric aggregates (clusters of telomeres that cannot be resolved further at an optical resolution limit of 200 nm), (4) nuclear volume, (5) *a/c ratio* (a spatial feature assessing cell cycle progression and proliferation), and (6) the spatial distribution of telomeres within the nuclear space, which reflects gene expression. Telomere dynamics were analyzed across cell cycle stages (G0/G1, S, and G2). Graphical representations were generated for subgroups of treated cells, illustrating telomere signal intensity, aggregate frequency, and signal distribution per cell. For telomere parameter comparisons (including total telomere number, aggregate count, signal intensity, *a/c ratio*, and nuclear volume), statistical analysis was performed using an unpaired two-tailed Student’s *t*-test between control and reversine-treated groups for each cell line (K-562 and MEG-01). Data are expressed as mean ± SD from at least 50 nuclei per condition, and *p*-values < 0.05 were considered statistically significant. To investigate the pro-apoptotic effects of reversine in chronic myeloid leukemia, we conducted a series of assays using the CML cell lines K-562 and MEG-01, which serve as well-established in vitro models for studying leukemic cell response.

### 2.6. Aurora Kinase mRNA Analysis

To investigate the modulation of *AURKA* and *AURKB* expression levels, after and before reversine treatment, quantitative real-time PCR (qPCR) was performed. Total RNA was extracted from treated and control cells using TRIzol^®^ reagent (Invitrogen, Carlsbad, CA, USA), and RNA purity was confirmed using a NanoDrop spectrophotometer (Thermo Fisher Scientific, Waltham, MA, USA) (260/280 ratio > 1.8). cDNA synthesis was carried out using the High-Capacity cDNA Reverse Transcription Kit (Applied Biosystems, Foster City, CA, USA). Quantitative PCR (qPCR) analysis was performed using SYBR^®^ Green Master Mix (Thermo Fisher Scientific, Waltham, MA, USA) on an ABI 7500 Real-Time PCR System (Applied Biosystems, Foster City, CA, USA). Specific primers for *AURKA*, *AURKB*, and *GAPDH* were used (Integrated DNA Technologies, Coralville, IA, USA). The primer sequences were as follows: *AURKA* (forward: 5′-TGTCAGTCTTGAGGCTGCTG-3′, reverse: 5′-TGTAGTTGTAGCCCTGGAGC-3′), *AURKB* (forward: 5′-GCTCTTCCGGTGTTTTGAGC-3′, reverse: 5′-CTGGGGTCTTCTTGGTCTTG-3′), and *GAPDH* (forward: 5′-GAAGGTGAAGGTCGGAGTCA-3′, reverse: 5′-GACAAGCTTCCCGTTCTCAG-3′). The reaction conditions included an initial denaturation at 95 °C for 10 min, followed by 40 cycles of 95 °C for 15 s and 60 °C for 1 min. The relative expression levels of *AURKA* and *AURKB* were determined using the 2^−ΔΔCt^ method, with control samples serving as the reference group. *GAPDH* was selected based on its validated stability across multiple leukemia studies and its consistent expression in CML-derived cell lines under drug treatment and stress conditions. Previous investigations have confirmed that *GAPDH* exhibits minimal variability in transcriptional levels in leukemic cells and remains one of the most reliable reference genes for gene expression normalization in hematologic malignancies [23,24,25].

Statistical analysis of all experiments was performed using GraphPad Prism 8 (GraphPad Software, San Diego, CA, USA). Differences between control and treated groups were analyzed using one-way ANOVA with Bonferroni post hoc tests for multiple comparisons. A *p*-value of less than 0.05 was considered statistically significant.

## 3. Results

**Figure 1:**
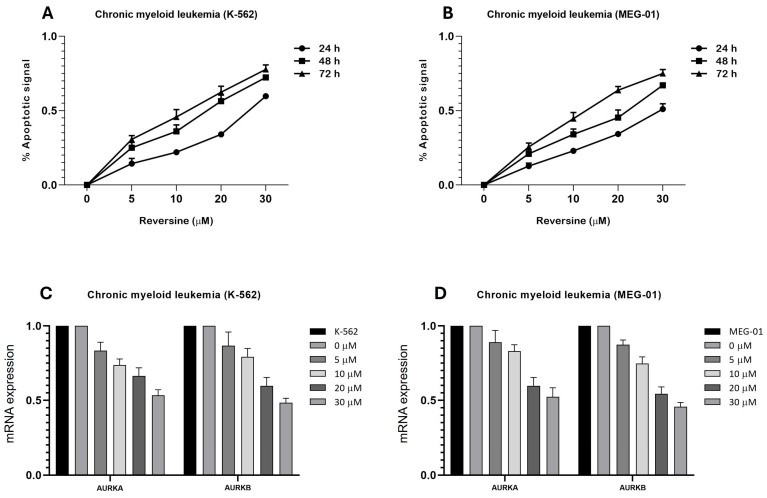
Reversine induces a time- and dose-dependent apoptotic response in Chronic Myeloid Leukemia (CML) cell lines and modulates aurora kinase gene expression. (**A**) K-562 and (**B**) MEG-01 cells were treated with increasing concentrations of reversine (5, 10, 20, and 30 µM) for 24 h (●), 48 h (∎), and 72 h (▲). Apoptotic activity was assessed using the Caspase-Glo^®^ 3/7 Assay System (Promega), and luminescence values were normalized to DMSO control (<0.1%). Data are presented as mean ± SD from three independent experiments performed in triplicate. Statistical analysis was performed using one-way ANOVA followed by Bonferroni post hoc test. (**C**,**D**) qPCR analysis of *AURKA* and *AURKB* mRNA expression in both cell lines treated with reversine (0, 5, 10, 20, and 30 µM) for 24 h relative to DMSO, which served as the calibrator sample. Data are shown as mean ± SD of three independent experiments. Where differences did not reach statistical significance, results are discussed as observed tendencies rather than significant effects.

To investigate the pro-apoptotic effects of reversine in CML cells, we analyzed caspase-3/7 activity in K-562 and MEG-01 treated with increasing concentrations of reversine (5, 10, 20, and 30 µM) for 24, 48, and 72 h. As shown in Figure 1A,B, a clear dose- and time-dependent apoptotic response was observed in both cell lines. In K-562 cells, caspase-3/7 activation increased steadily with both dose and treatment duration. The apoptotic signal nearly doubled from 24 to 72 h at intermediate doses (10–20 µM), while treatment with 30 µM reversine led to ~0.55 apoptotic signal at 24 h, rising to ~0.80 by 72 h, suggesting an enhancement of reversine efficacy over time. At lower doses (5 µM), apoptotic signals remained modest, indicating a threshold effect (Figure 1A). For MEG-01 cells, the apoptotic pattern was also pronounced at 72 h. At 30 µM, caspase activity reached also approximately 0.75 at 72 h, while the 48 h time point yielded ~0.65, and 24 h stayed near 0.5. This supports a strong time-dependent trend (Figure 1B).

Nonlinear regression analysis yielded the following half-maximal apoptotic dose (DL_50_) values for both cell lines were K-562 cells, 18.73 µM (48 h), and for MEG-01 cells 22.55 µM (48 h). These findings confirm a progressive reduction in DL_50_ with increased treatment time, indicating enhanced apoptosis with prolonged exposure. Collectively, these results highlight reversine’s potential as a time- and dose-sensitive pro-apoptotic agent in CML models, with stronger effects in K-562 cells at earlier timepoints and a delayed but robust response in MEG-01.

Based on the literature and our experimental findings, reversine appears to induce apoptosis in CML cell lines through activation of caspase-3/7 and upregulation of Fas and DR5 receptors, consistent with previous studies [26,27,28]. These results support its role as a pro-apoptotic agent with potential therapeutic applications.

Additionally, we observed a significant downregulation of *AURKA* and *AURKB* mRNA levels following reversine treatment. This effect is likely mediated by reversine-induced mitotic failure and cellular dedifferentiation, which reduces the demand for mitotic regulators and represses their transcription, as similarly described in acute leukemia models. Quantitative PCR analysis assessed the relative expression levels of *AURKA* and *AURKB* mRNA after 24 h of reversine treatment (Figure 1C,D).

To investigate the phenotypic effects of reversine, based on telomere dynamics in CML cell lines, we employed the half-maximal apoptotic dose (DL_50_) as the treatment reference. As previously determined, after nonlinear regression analysis, the DL_50_ values were 18.73 µM (48 h) for K-562 cells and 22.55 µM (48 h) for MEG-01 cells. These concentrations were used to ensure a controlled level of cytotoxicity while preserving a viable cell population suitable for phenotypic and structural analysis. Reversine has been previously reported to induce cellular dedifferentiation through disruption of mitotic spindle checkpoints and the enhancement of stem cell-like phenotypes, thereby serving as a valuable tool for investigating nuclear reprogramming and genomic plasticity in leukemic cells.

The three-dimensional (3D) architecture of nuclear telomeres was evaluated using TeloView^®^ software [16,17], which enables quantitative analysis of telomere distribution, clustering (aggregates), signal intensity, *a/c ratio*, and nuclear volume. This approach allowed us to assess how reversine-driven dedifferentiation alters telomere organization and nuclear architecture in CML cells. The use of DL_50_-based treatments provided a robust model for observing sublethal, yet biologically significant, changes in telomere dynamics associated with early apoptotic commitment and cellular reprogramming (Table 1).

**Figure 2:**
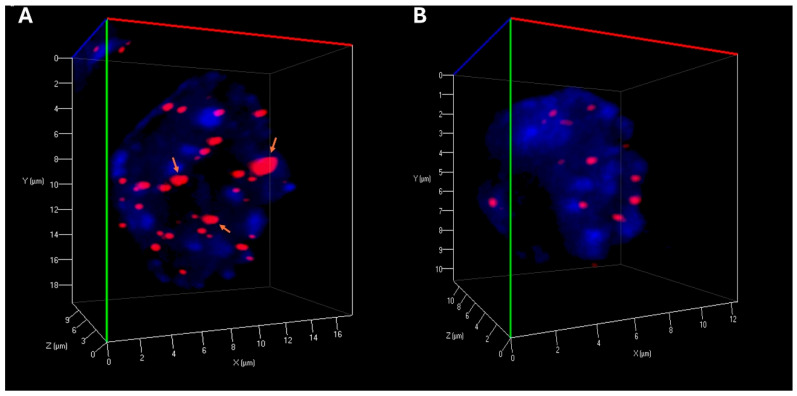
Three-dimensional Telomere Architecture in CML Cells. (**A**) Representative 3D nuclear telomere distribution (red) within the counterstained nucleus (blue) in untreated MEG-01 cells. Orange arrows highlight telomere aggregates. (**B**) 3D telomere distribution in MEG-01 cells treated with reversine, showing a reduced frequency of telomere aggregates.

Our experiments revealed important alterations in telomere organization following reversine exposure to CML cells. The total number of telomeres was reduced, suggesting telomere reorganization, potentially reflecting decreased chromosomal instability. The number of telomere aggregates was also significantly lower in treated cells, indicating reduction in telomere clustering, which may be linked to impaired chromatin architecture and genome stability (Table 1) (Figure 2). Moreover, the *a/c ratio*, a critical parameter reflecting the spatial organization of telomeres and nuclear shape, exhibited modifications, suggesting an impact of reversine on nuclear integrity. Additionally, nuclear volume was markedly reduced in both K-562 and MEG-01 cells, reinforcing the hypothesis that reversine induces nuclear condensation and structural reorganization (Table 1).

From the perspective of genomic instability, we observed that reversine was able to promote cellular changes, possibly by disrupting mitotic spindle checkpoints and inducing a stem-like phenotype. Our results suggest a cellular state characterized by reduced genomic instability and a “less aggressive” profile. During the reprogramming of somatic cells to a “pluripotent state”, significant chromatin remodeling occurs, which could affect telomere structure and organization. The reprogramming processes can lead to telomere maintenance, depending on the cellular context and stage of dedifferentiation. However, it is important to consider that our model is based on established cell lines; therefore, it will be necessary to confirm these phenotypic changes in patient-derived samples in subsequent studies.

**Figure 3:**
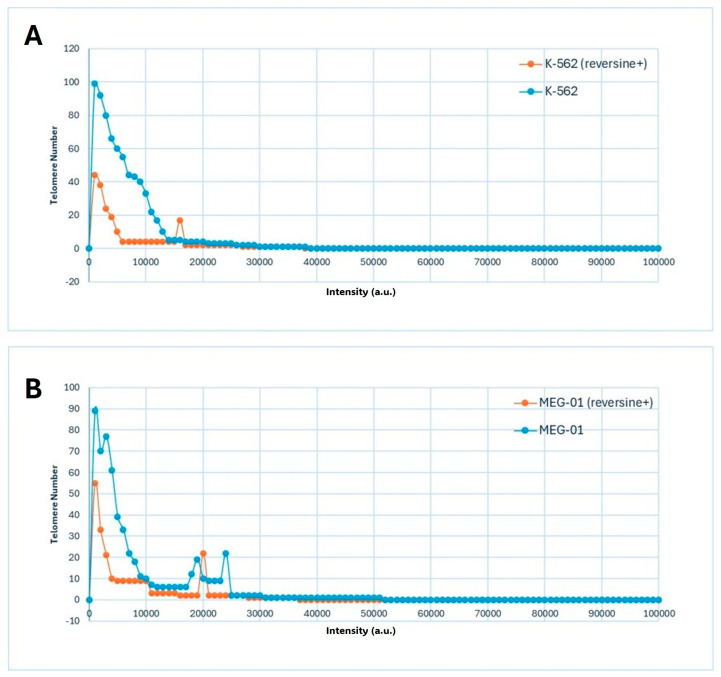
Distribution of telomere signal intensities in Chronic Myeloid Leukemia (CML) cell lines before and after reversine treatment. (**A**) K-562 and (**B**) MEG-01 cells were analyzed using 3D quantitative fluorescence in situ hybridization (Q-FISH) followed by TeloView^®^ processing. Each graph depicts the frequency distribution of telomere signal intensities, which correlate with relative telomere length, for untreated (control) and reversine-treated samples. Reversine exposure resulted in a global shift toward lower signal intensities, reflecting a reduction in telomere number and overall fluorescence per nucleus, consistent with telomere reorganization and chromatin condensation observed in Table 1 and Figure 2.

The data presented in Figure 3A,B illustrated the distribution of telomere intensity, a parameter correlating with telomere length, in CML cells under distinct experimental conditions. The analysis included two CML cell lines, K-562 and MEG-01, each assessed in both untreated and reversine-treated states.

The graphical representation demonstrated a three-dimensional telomere profiling approach, capturing variations in telomere length distribution across the different conditions (Figure 3A,B). In untreated cells, the telomere intensity distribution exhibited a characteristic pattern, indicative of a heterogeneous population with varying telomere lengths (Figure 3A,B). Upon reversine treatment, shifts in this distribution were observed, suggesting an influence on telomere integrity or regulatory mechanisms governing telomere length maintenance.

## 4. Discussion

The findings of this study provide evidence that reversine, a known aurora kinase inhibitor, significantly alters the three-dimensional (3D) telomere architecture in CML cell lines, specifically K-562 and MEG-01. These modifications in telomere dynamics following reversine treatment may represent a mechanistic pathway through which the compound exerts its antileukemic effects. In addition, reversine is also known to induce a dedifferentiated cellular phenotype, promoting a more plastic and progenitor-like state. This dedifferentiation effect can contribute to genomic reorganization, including nuclear remodeling and altered telomere positioning, further enhancing cellular susceptibility to mitotic stress and apoptosis [3,29,30].

Telomeres, the protective caps at the ends of chromosomes, play a crucial role in maintaining genomic stability. In CML, telomere dysfunction has been associated with disease progression and poor prognosis [19,31]. Studies have demonstrated that as CML progresses from the chronic phase to blast crisis, telomere shortening becomes more pronounced and is closely associated with increased genomic instability [32,33]. In contrast to the natural progression of CML, our cell line model employed a ‘phenotype reversion strategy’ using reversine at concentrations near the DL_50_, aiming to induce cellular reprogramming rather than disease advancement. Reversine treatment resulted in marked alterations in telomere length and three-dimensional spatial organization. These changes are more plausibly attributed to apoptotic mechanisms, mitotic stress, and nuclear remodeling, rather than classical dedifferentiation alone. While reversine has been shown to induce a progenitor-like phenotype, its mode of action involves inhibition of mitotic kinases, leading to chromatin compaction, polyploidy, and cell death. The decrease in telomere signal intensity and number observed in our study may reflect telomere uncapping or degradation secondary to nuclear condensation or early apoptotic signaling, as described in mitotic arrest models [34,35]. Although dedifferentiation in stem cell models is typically associated with telomere elongation via telomerase activation, this mechanism does not apply directly to reversine’s cytotoxic action in leukemic cells, which already express telomerase and are subject to intense mitotic disruption. Therefore, we propose that reversine-induced nuclear remodeling and telomeric alterations represent a combined consequence of chromatin instability and early apoptotic commitment rather than telomere maintenance reprogramming [36,37].

Interestingly, while dedifferentiation is often associated with increased cellular plasticity, reversine may contribute to telomere and chromatin stability in CML cells. This suggests the possibility of a reduced cytotoxic impact on surviving cells rather than necessarily driving a shift toward a less differentiated state. Additionally, the notable reduction in nuclear volume observed in this study could imply that reversine influences nuclear compaction, a phenomenon that is frequently linked to apoptosis or chromatin condensation resulting from mitotic arrest [30,38].

The reduction in telomere aggregates observed following reversine treatment suggests that the compound interferes with telomere clustering, a structural phenomenon linked to nuclear disorganization and chromosomal instability in cancer cells [30,39,40]. As telomere organizations are a critical component of genomic architecture, this remodeling may represent a shift toward a more stable and less proliferative nuclear state. Additionally, we observed a significant decrease in telomere signal intensity, which may reflect telomere shortening. While telomere attrition is typically associated with replicative senescence or progression to more aggressive disease phases, particularly in CML, it is important to contextualize this finding within our experimental model. Our study was conducted using aneuploid CML cell lines characterized by baseline telomere disorganization and high aggregate frequency. Thus, the telomere shortening seen here likely reflects a treatment-induced remodeling process, potentially linked to early cell cycle arrest or apoptotic signaling, rather than disease progression. Indeed, telomere dysfunction, particularly when accompanied by decreased aggregation and reduced nuclear volume, may contribute to cell cycle exit and apoptosis, consistent with reversine’s dual action as a mitotic inhibitor and dedifferentiation agent [2,3]. These observations underscore reversine’s capacity to induce structural and functional reprogramming of the leukemic genome, supporting its therapeutic relevance in models of genomic instability.

To comprehensively analyze the effects of reversine on telomere dynamics, we utilized TeloView^®^ software [16,17], an advanced imaging and analytical tool that enables high-throughput 3D telomere analysis. TeloView^®^ quantitatively assesses telomere organization within interphase nuclei, measuring parameters such as total telomere number, telomere signal intensity, aggregate formation (clustering of telomeres), *a/c ratio* (a measure of chromatin and cell cycle distribution), and nuclear volume. In cancer research, TeloView^®^ has been instrumental in identifying telomeric signatures associated with genomic instability, disease progression, and response to therapy [16,17,18,19].

On the other hand, Aurora kinases—particularly *AURKA* and *AURKB*—are pivotal regulators of mitosis and have been implicated in the pathogenesis of various malignancies, including CML [5,19,41]. Their overexpression has been associated with increased tumor cell proliferation and survival [42,43,44]. Reversine’s inhibitory effect on these kinases, under determined concentrations, may impair mitotic progression, leading to mitotic defects and apoptosis in leukemic cells [13,45]. This proposed mechanism is further supported by studies demonstrating that aurora kinase inhibition can trigger mitotic catastrophe and promote cell death in cancer models [46,47,48].

Utilizing three-dimensional telomere analysis via the TeloView^®^ platform allowed for a more precise evaluation of telomere organization within the nuclear architecture. This detailed analysis not only linked reversine treatment to the inhibition of mitotic progression but also highlighted its role in inducing cellular changes. This advanced methodological approach allowed for the assessment of spatial distribution alongside telomere length heterogeneity, facilitating a comprehensive understanding of the impact of reversine on telomere biology in CML cells. These findings may have implications for therapeutic strategies targeting telomere-associated mechanisms in leukemia.

The clinical implications of these findings are significant. Targeting telomere dynamics and aurora kinase activity presents a promising therapeutic strategy in CML. Telomerase inhibitors have already shown potential in the treatment of myeloid malignancies [49,50,51], and the addition of aurora kinase inhibitors like reversine could enhance therapeutic efficacy by simultaneously disrupting multiple pathways critical for leukemic cell survival [52,53,54,55]. Moreover, the ability of reversine to induce changes in 3D telomere architecture may serve as a biomarker for treatment response, aiding in the stratification of patients and the optimization of therapeutic regimens.

In conclusion, this study underscores the multifaceted role of reversine in modulating telomere dynamics and Aurora kinase activity in CML cells. The integration of TeloView^®^ for 3D telomere analysis provided valuable insights into the structural and functional changes induced by reversine, highlighting its potential as a therapeutic agent in the management of CML.

## Figures and Tables

**Table 1:** Quantitative analysis of 3D telomere parameters in Chronic Myeloid Leukemia (CML) cell lines (K-562 and MEG-01) before and after reversine treatment. Data represent mean ± SD from ≥50 interphase nuclei per condition, analyzed using unpaired two-tailed Student’s *t*-test (*p* < 0.05).

CML Cells	Total Number of Telomeres (Mean ± SD)	Total Number of Telomere Aggregates (Mean ± SD)	Total Intensity (Mean ± SD)	Intensity of All Signals (Mean ± SD)	*a/c ratio* (Mean ± SD)	Nuclear Volume (Mean ± SD)
K-562	56.27 ± 17.88	10.21 ± 3.89	643.123 ± 231.337	39.543 ± 2562	17.88 ± 2.97	547,621 ± 176,234
K-562 (reversine+)	22.39 ± 11.82	4.67 ± 2.65	433.332 ± 233.312	28.543 ± 2984	11.44 ± 3.89	266,431 ± 134,654
*p* value	*p* < 0.0001	*p* < 0.0001	*p* < 0.0001	*p* < 0.0001	*p* < 0.0001	*p* < 0.0001
MEG-01	45.22 ± 12.21	9.44 ± 3.88	543.341 ± 189.132	26.876 ± 3241	16.43 ± 2.33	453,765 ± 176,432
MEG-01 (reversine+)	29.33 ± 9.67	5.55 ± 2.86	312.133 ± 109.102	19.432 ± 2987	9.66 ± 2.32	278,543 ± 131,762
*p* value	*p* < 0.0001	*p* < 0.0001	*p* < 0.0001	*p* < 0.0001	*p* < 0.0001	*p* < 0.0001

## Data Availability

The datasets generated and analyzed during the current study are available from the corresponding author upon reasonable request. All data supporting the conclusions of this article are included within the manuscript.
